# Minimal Clinically Important Difference on Parkinson's Disease Sleep Scale 2nd Version

**DOI:** 10.1155/2015/970534

**Published:** 2015-10-11

**Authors:** Krisztina Horváth, Zsuzsanna Aschermann, Péter Ács, Gabriella Deli, József Janszky, Sámuel Komoly, Kázmér Karádi, Márton Kovács, Attila Makkos, Béla Faludi, Norbert Kovács

**Affiliations:** ^1^Doctoral School of Clinical Neuroscience, University of Pécs, Rét Utca 2, Pécs 7623, Hungary; ^2^Department of Neurology, University of Pécs, Rét Utca 2, Pécs 7623, Hungary; ^3^MTA-PTE Clinical Neuroscience MR Research Group, Rét Utca 2, Pécs 7623, Hungary

## Abstract

*Background and Aims*. The aim of the present study was to determine the estimates of minimal clinically important difference for Parkinson's Disease Sleep Scale 2nd version (PDSS-2) total score and dimensions. *Methods*. The subject population consisted of 413 PD patients. At baseline, MDS-UPDRS, Hoehn-Yahr Scale, Mattis Dementia Rating Scale, and PDSS-2 were assessed. Nine months later the PDSS-2 was reevaluated with the Patient-Reported Global Impression Improvement Scale. Both anchor-based techniques (within patients' score change method and sensitivity- and specificity-based method by receiver operating characteristic analysis) and distribution-based approaches (effect size calculations) were utilized to determine the magnitude of minimal clinically important difference. *Results*. According to our results, any improvements larger than −3.44 points or worsening larger than 2.07 points can represent clinically important changes for the patients. These thresholds have the effect size of 0.21 and −0.21, respectively. *Conclusions*. Minimal clinically important differences are the smallest change of scores that are subjectively meaningful to patients. Studies using the PDSS-2 as outcome measure should utilize the threshold of −3.44 points for detecting improvement or the threshold of 2.07 points for observing worsening.

## 1. Introduction

The nonmotor symptoms of Parkinson's disease (PD) have been increasingly recognized as major burden of quality of life [1, 2]. Sleep-related problems are one of the most frequent and troublesome nonmotor aspects of PD. Sleep-related problems are certainly multidimensional. The recently developed Parkinson's Disease Sleep Scale 2nd version (PDSS-2) was designed to be simultaneously able to capture the multidimensional aspects of sleep-related problems and any changes in sleep quality [3]. It consists of 15 items evaluating three domains (motor symptoms at night, PD symptoms at night, and disturbed sleep) [3]. Symptoms on each domain can be scored in the range of 0–20 points, higher scores representing more nighttime sleep-related problems. The sum of the three domains gives the total score of PDSS-2 with the maximum value of 60 points. PDSS has been translated and validated into several languages [4, 5] and has good clinical validity [6]. The threshold indicating sleep problems is 11 points for the Hungarian version of PDSS-2 [7].

Even though the PDSS-2 has been utilized in several pharmacological [8–12] and neurosurgical [13] studies to identify any improvement in nocturnal sleep quality, the magnitude of change required to represent a clinically meaningful improvement has not been evaluated yet.

One of the major issues in recent biomedical research is the evaluation of clinical meaning of changes on patients' reported outcomes (PROs). As some changes may be statistically significant but clinically irrelevant, statistical significance does not necessarily imply clinical importance: interventions with small effect size, for example, may have no clinical importance for the patients or clinicians.

To overcome this issue, the concern of minimal clinically important difference (MCID) was introduced in late 1980s. Jaeschke et al. defined the MCID as “the smallest difference in score in the domain of interest which patients perceive as beneficial and which would mandate…a change in the patient's management” [14]. In other words, MCID is the smallest change in an outcome measure that a patient would identify as important. Therefore, MCID offers a threshold above which outcome is experienced as relevant by the patient avoiding the problem of mere statistical significance.

Evaluation of MCIDs for different outcome measures not only is important because of clinical decision-making and the labeling claims of medical products but also is required for study design as it is essential for calculating the sample sizes required for different trials and surveys.

However, there are some important issues with the MCID thresholds. First, thresholds for detecting minimal clinically meaningful improvement and worsening may be asymmetric. Therefore, different threshold values may exist for detecting improvement and deterioration on the same outcome measure [15].

A more troublesome concern is the methodology- and sample-dependent nature of MCID. At the moment numerous different approaches are available for MCID calculations (e.g., anchor-based and distribution-based techniques). For example, application of different methods even on the same sample can result in different MCID values [15, 16]. On the contrary, the usage of the same outcome measures and methods for MCID calculation on different study population can also yield different MCID thresholds [15, 17]. To overcome these problems, an article summarizing the recommendations on methods for evaluating MCID was recently published [18]. According to its proposals, the estimation of MICD for a specific outcome measure should be based on multiple approaches. Because the PRO measures should correlate with the appropriate clinical anchor used for determining MCID, the value of correlation coefficient should be at least 0.3 between them.

The aim of the present study was to determine the estimates of MCID for Parkinson's Disease Sleep Scale 2nd version (PDSS-2) in a longitudinal observational setup. Our protocol fully complied with the recommendations for determining MCIDs [18] and simultaneously multiple techniques were assessed for the calculations.

## 2. Materials and Methods

### 2.1. Patients

In this prospective study 413 consecutive patients fulfilling the UK Brain Bank criteria for PD were enrolled [19] at Department of Neurology, University of Pécs, Hungary, between 2013 and 2015. The patients were examined by neurologists specialized in movement disorders. Each subject gave written informed consent in accordance with the ethical approval of Regional and Institutional Ethical Committee (3617.316-24987/KK41).

### 2.2. Obtained Rating Scales

Besides PDSS-2 sociodemographic and PD-related data were obtained and the patients were screened for dementia by the means of Montreal Cognitive Assessment and Mattis Dementia Rating Scale [20] at baseline. Patients with atypical parkinsonism or dementia (receiving ≤125 points on Mattis scale and/or fulfilling the criteria of DSM-5 for major neurocognitive disorder in PD [21, 22]) were excluded from the study. Severity of Parkinson's disease was assessed by the Hungarian validated version of Movement Disorders Society-Sponsored Unified Parkinson's Disease Rating Scale (MDS-UPDRS) [23, 24], the Hoehn-Yahr Scale (HYS) [25], and the Schwab-England Scale (SES) [26]. Implying the recommendations of the Movement Disorders Society Task Force, the original HYS was obtained and treated as ordinal values [27]. Baseline characteristics of the study population are demonstrated in Table 1.

Patients were reexamined 9 months (275 ± 21 days) later after receiving standard clinical care. After completing the PDSS-2 questionnaire, the patients were asked to describe if their sleep quality was either “very much better,” “much better,” “a little better,” “the same,” “a little worse,” “much worse,” or “very much worse” since the last visit. These answers were identical with the Hungarian validated version of Patient-Rated Global Impression of Improvement (PGI-I) items adjusted for sleep problems.

### 2.3. Anchor-Based Methods to Determine MCID

The anchor-based approaches utilize either patient-based or clinician-based external indicator to assign subjects into several groupings reflecting no change, small negative changes, large negative changes, small positive changes, or large positive changes. Two different types of anchor-based approaches were applied to determine CIDs. These types of methods require an independent standard or anchor that is simultaneously interpretable by itself, clinically relevant, and correlated with the instrument being evaluated [28, 29]. For this study, the above-mentioned PGI-I items served as the anchors for calculating the MCID estimates for PDSS-2.

#### 2.3.1. Within-Patients Score Change Method

This approach defines MCID as the change between the PRO scores of a group of patients selected according to their answer to a global assessment scale (anchor). Therefore, in this study we calculated the mean change on the PDSS-2 dimensions and the total score for those subjects who indicated “no change” at follow-up or for those who indicated “a little worse” or “a little better” change.

#### 2.3.2. Sensitivity- and Specificity-Based Approach

This second anchor-based method is useful in calculating the threshold that allows for the best discrimination between groups of patients. For example, the score that produces the greatest sensitivity and specificity for discriminating patients with minimal change from patients without any change can be considered as the MCID. Generally sensitivity is the proportion of subjects with a positive test out of the group of subjects who were truly positive. Likewise, specificity is the proportion of subjects with a negative test out of the group of subjects who were truly negative. Used in conjunction with MCID estimations, sensitivity is the proportion of the patients who report a change on the external criterion (i.e., PGI-I) and whose PRO score (e.g., PDSS-2) change exceeds the threshold MCID value. Similarly, specificity is the proportion of subjects who do not report a change on the external criterion (anchor) and whose PRO score changes are below the threshold MCID value. A sensitivity of 100% indicates that all true positives are identified, whereas a specificity of 100% indicates that all the true negatives are identified.

In our study, we applied receiver operating characteristic (ROC) curve technique to find the most suitable MCID values. Because the recommendations for desirable MCID sensitivity and specificity levels have yet to be determined [18], we followed the method described by Hauser et al. [15, 17]. Assuming that false-positive and false-negative identifications are equally unwanted, we determined the cutoff value with the most optimal balance between sensitivity and specificity. The optimal cutoff points to distinguish changes on PDSS-2 between subjects rated as minimally worsened or minimally improved and subjects rated as unchanged on the PGI-I score were estimated as the point on the ROC curve closest to the point of (0,1). It was calculated as the minimum value of the square root of (1 − sensitivity)^2^ + (1 − secificity)^2^. For the most optimal cutoff values the positive (LR+) and negative (LR−) likelihood-ratios were also determined using the following formulas:

(1) LR+True  positive  rateFalse  positive  rate=Sensitivity1−Specificity,LR−False  negative  rateTrue  negative  rate=1−SensitivitySpecificity.

### 2.4. Distribution-Based Method to Determine MCID

The distribution-based methods compare the changes in PRO scores to some measure of variability. However, the distribution-based estimates provide no direct information about the MCID. They are simply a way of describing the observed differences in a standardized metric [18].

Effect size is generally a measure of exactly how strong the relationship that was being examined is. Common effect sizes are mean differences, correlation coefficients, regression coefficients, odds ratios, and hazard ratios. The value of the effect size represents the number of standard deviations (SDs) by which the scores have changed from baseline to the follow-up. By convention, an effect size of 0.2 is considered as small, 0.5 as moderate, and 0.8 as large [18]. Used in conjunction with anchor-based methods, effect size ascertains the responsiveness of the external criterion. With regard to MCID, for example, the change in scores corresponding to small effect size should estimate the MCID value [18, 29].

### 2.5. Statistical Analysis

All statistical analyses were carried out using IBM SPSS software package (version 21, SPSS Inc., Chicago, USA). We calculated Spearmen's correlation coefficients to assess the relationship between the PGI-I and the changes in PDSS-2 scores. Comparison of baseline and follow-up scores was performed by paired *t*-tests. Statistical significance level was set to 5%. Because the SPSS Suite did not have built-in functions for calculating positive and negative predictive values, we utilized the syntax available on the IBM website (http://www-01.ibm.com/support/docview.wss?uid=swg21483380, assessed on Jan 15, 2013).

## 3. Results

During the observational period the levodopa dose in LED increased from 585.4 ± 472.1** **mg to 735.3 ± 490.4 mg and the dopamine-agonist dose (measured in LED) increased from 215.6 ± 244.9** **mg to 323.2 ± 234.4 mg, whereas the number of patients on dopamine-agonist therapy increased from 165 to 324 (Table 1).

### 3.1. Anchor-Based MCID Estimation

The Spearman correlation coefficient assessing the correlation between the PGI-I and the change in PDSS-2 was 0.364 (*p* < 0.001). As a correlation coefficient higher than 0.3 between the anchor and the PRO is required for detecting MCID [18], our study setting can be considered as a suitable dataset for detecting MCID for PDSS-2.

#### 3.1.1. Within-Patients Score Change Method

Because we aimed to determine only the magnitude of minimal clinically important difference, only the data for those judged minimally improved (*n* = 142), unchanged (*n* = 126), and minimally worse (*n* = 154) are presented in Table 2.

Mean changes (±SD) for PDSS-2 for subjects rated minimally improved, unchanged, or minimally worse on PGI-I scale are demonstrated in Table 2. The mean change for patients rating the same sleep quality was −0.54 (±3.24), whereas for minimal improvement it was −3.44 (±6.40) and for minimal worsening it was 2.07 (±7.72) points on the total score of PDSS-2 (Table 2).

#### 3.1.2. Sensitivity- and Specificity-Based Approach

Subsequently we performed ROC analysis between the changes in the total score of PDSS-2 compared and the PGI-I as state variable. The most optimal cutoff value discriminating the minimal improvement was ≤−3 points on the total score of PDSS-2, whereas the best cutoff to identify the minimal worsening was ≥2 points (Table 3).

### 3.2. Effect Size Method

The estimates calculated by anchor-based methods for detecting minimal clinically meaningful improvement and worsening represent the effect size of 0.21 and −0.21, respectively.

Because both anchor-based and distribution-based calculations gave similar results, we could estimate that the threshold representing minimal clinically important difference for improvement was −3.44 points and for worsening it was +2.07 points.

## 4. Discussion

Following the recommendations of Revicki et al. [18], our aim was to evaluate the magnitude of minimal clinically important difference on PDSS-2. By the utilization of the combination of both anchor- and distribution-based methods, we were able to estimate the MCID thresholds for the total score of PDSS-2 congruently. Based on our results, the magnitude of MCID is asymmetric for improvement and worsening. According to our results, any improvement larger than −3.44 points and any worsening larger than +2.07 points can represent clinically important changes for the patients. This asymmetry is probably due to the asymmetric perception of sleep quality. According to our data relatively larger improvements are required to be judged by the patients as positive improvement, whereas a relatively smaller worsening can elicit the perception of worsening.

One of the limitations of our study may be that we utilized only patient-derived anchors for assessing MICD and our data is not based on the objective (e.g., polysomnographic or actigraphic) findings. Because sleep quality is very subjective, in our opinion the severity of sleep problems can only reliably be described by the patients. There is no objective physical or instrumental examination which could reliably measure the sleep quality of the patients. Not even the sleep labs can describe all dimensions of the sleep. Polysomnography (PSG), for example, can objectively detect the presence and measure the severity of PMLS in an artificial setup, but it is still unable to capture several other aspects of sleep quality [30, 31]. Although the PDSS was validated against PSG [30, 31], not even the original (English) version of PDSS-2 was validated against any sleep lab tests [3]. This was the reason why we applied a patient-derived (and not a clinician-based) anchor to assess the MICD. Because similar approach was utilized for pain outcomes [32], our method is acceptable for detecting MICD for PDSS-2.

Since its publication in 2011 [3], a growing number of studies utilized the PDSS-2 to evaluate changes in sleep problems of PD patients. However, in the lack of MICD value for PDSS-2, these studies could demonstrate only statistical significance and not clinically meaningful difference.

The first larger study aimed to investigate the effects of rotigotine on nocturnal sleep quality [33]. This was the first study where early morning motor function and nocturnal sleep-disturbances served as the coprimary endpoints. In this double-blind study, the mean PDSS-2 total score had decreased by −5.9 points with rotigotine and by −1.9 points with placebo. Because the difference between the active and placebo arm (−4.0) is larger than our MCID value (−3.56), the observed difference can be considered as clinically meaningful.

Zibetti et al. demonstrated that 2–4 months' long levodopa/carbidopa intestinal gel (LCIG) treatment can improve the nighttime sleep quality in PD measured by PDSS-2 (improvement from 34.0 points to 20.9 points) [10]. Similar efficacy was observed by Kovács et al. [12]. They observed 7-point improvement (from 25 points to 18 points) on PDSS-2 total score after 6-month long LCIG treatment [12].

Recently, Deli et al. demonstrated the beneficial effects of bilateral subthalamic deep brain stimulation on the sleep quality in PD. In that study the total score of PDSS-2 decreased from 24 (median, IQR: 17–32) to 10 (median, IQR: 7–18) points (*p* < 0.001) in the population of 25 advanced PD patients [13]. Meanwhile, the number of patients having clinically troublesome sleep problems also decreased from 13 to 3. Based on our MCID estimations, all of these reported changes are also clinically meaningful.

## 5. Conclusions

Minimal clinically important differences are the smallest change of scores that are subjectively meaningful to patients. The results of our study estimate the minimum magnitude of change that should be sought when studies are designed using the PDSS-2 to evaluate the change over time in the sleep quality in PD. Studies using the PDSS-2 as outcome measure should utilize the threshold of −3.44 points for detecting improvement or the threshold of +2.07 points for observing worsening.

## Figures and Tables

**Table 1:** The demographic and disease-specific characteristics of the study population (*n* = 413) at baseline examination.

	Mean or number of patients	Standard deviation	Median	Percentile 25	Percentile 75
Age	64.83	9.20	65.00	59.00	72.00
Sex	285M/128F				
Education (years)	11.9	3.4	12.0	11.0	16.0
Disease duration	9.91	5.99	9.00	5.00	13.00
HYS (number of patients at stage 1/2/3/4/5)	68/125/170/35/15				
Patients on levodopa (baseline)	215 (52.1%)				
Patients on levodopa (follow-up)	265 (64.2%)				
Levodopa dosage (baseline, in LED, mg)	585.4	472.1	520.0	200.0	812.5
Levodopa dosage (follow-up, in LED, mg)	735.3	490.4	620.0	350.0	912.5
Patients on dopamine-agonists (baseline)	165 (39.9%)				
Patients on dopamine-agonists (follow-up)	324 (78.5%)				
Dopamine agonist dosage (baseline, in LED, mg)	215.6	244.9	160.0	.0	320.0
Dopamine agonist dosage (follow-up, in LED, mg)	323.2	234.4	324.0	98.5	420.0
Patients on benzodiazepines (both baseline and follow-up)	44 (10.7%)				
Schwab-England	74.2	13.9	80.0	70.0	90.0
MDS-UPDRS nMEDL	14.8	6.5	14.0	10.0	19.0
MDS-UPDRS MEDL	17.0	8.3	16.0	10.0	23.0
MDS-UPDRS ME	40.3	14.8	39.0	29.0	48.0
MDS-UPDRS MC	5.2	3.6	5.0	2.0	7.0
MDS-UPDRS total score	77.2	25.8	74.0	58.0	91.0
PDSS-2 motor symptoms domain	4.7	3.9	5.0	2.0	7.0
PDSS-2 PD symptoms domain	3.6	3.3	4.0	1.0	5.0
PDSS-2 disturbed sleep domain	7.3	4.1	7.0	4.0	10.0
PDSS-2 total score	15.6	9.6	16.0	8.0	21.0

HYS: Hoehn-Yahr Stages; LED: levodopa equivalent dosage; MDS-UPDRS: Movement Disorders Society Sponsored Version of Unified Parkinson's Disease Rating Scale; MDS-UPDRS MC: Motor Complications (Part 4 of MDS-UPDRS); MDS-UPDRS ME: Motor Examination (Part 3 of MDS-UPDRS); MDS-UPDRS MEDL: Motor Experiences of Daily Living (Part 2 of MDS-UPDRS); MDS-UPDRS nMEDL: Non-Motor Experiences of Daily Living (Part 1 of MDS-UPDRS).

**Table 2:** Mean changes of PDSS-2 scores with respect to Patient-Rated Global Impression of Improvement scores.

	PDSS-2 total score					PDSS-2 motor symptoms domain					PDSS-2 PD symptoms domain					PDSS-2 disturbed sleep domain				
	Mean	SD	Median	25th percentile	75th percentile	Mean	SD	Median	25th percentile	75th percentile	Mean	SD	Median	25th percentile	75th percentile	Mean	SD	Median	25th percentile	75th percentile
PGI-I (sleep problems)																				
A little better	−3.44	6.40	−3.00	−5.00	2.00	−.92	2.98	0.00	−2.00	1.00	−.92	2.48	0.00	−1.00	0.00	−1.60	2.87	−2.00	−3.00	1.00
The same	−0.54	3.24	−1.00	−3.00	3.00	−.15	4.02	0.00	−3.00	2.00	−.15	3.38	0.00	−2.00	1.00	−.21	3.35	0.00	−3.00	1.00
A little worse	2.07	7.72	2.00	0.00	7.00	0.53	4.01	1.00	−1.00	3.00	0.47	3.62	0.00	−1.00	3.00	1.07	3.12	1.00	1.00	4.00

SD: standard deviation; PDSS-2: Parkinson's Disease Sleep Scale 2nd version; PGI-I: Patient-Rated Global Impression of Improvement.

**Table 3:** Best cutoff values discriminating minimal clinically important differences for PDSS-2 determined by receiver operating characteristic analysis.

	ROC curve cutoff	Sensitivity	Specificity	+LR	−LR	AUC	Significance
PGI-I							
A little better	<−3	54.00	51.40	1.11	0.89	0.523	0.046
The same	NA						
A little worse	≥2	50.98	65.42	1.47	0.75	0.613	0.023

AUC: area under the curve; +LR: positive likelihood-ratio; −LR: negative likelihood-ratio; PDSS-2: Parkinson's Disease Sleep Scale 2nd version; PGI-I: Patient-Rated Global Impression of Improvement; ROC: receiver operating characteristic analysis.

## Abbreviations

CID: Clinically important difference
HYS: Hoehn-Yahr Stage
LED: Levodopa equivalent dosage
LCIG: Levodopa/carbidopa intestinal gel
LR: Likelihood-ratio
MCID: Minimal clinically important difference
MDS-UPDRS: Movement Disorders Society Sponsored Version of Unified Parkinson's Disease Rating Scale
MDS-UPDRS MC: Motor Complications (Part 4 of MDS-UPDRS)
MDS-UPDRS ME: Motor Examination (Part 3 of MDS-UPDRS)
MDS-UPDRS MEDL: Motor Experiences of Daily Living (Part 2 of MDS-UPDRS)
MDS-UPDRS nMEDL: Non-Motor Experiences of Daily Living (Part 1 of MDS-UPDRS)
PD: Parkinson's disease
PDSS-2: Parkinson's Disease Sleep Scale 2nd version
PGI-I: Patient-rated Global Impression of Improvement
PRO: Patients' reported outcome
ROC: Receiver operating characteristic
SD: Standard deviation
SEM: Standard error of measurement
SES: Schwab-England Scale.
